# Fracture Risk Evaluation of Trochlear Groove Depth in Toy and Small-Breed Dogs Under Gait-Based Loading: A Finite Element and Fatigue Analysis Study

**DOI:** 10.3390/ani16132081

**Published:** 2026-07-05

**Authors:** Minuk Jeong, Heung-Myoung Woo, Kihoon Kim, Junhyung Kim

**Affiliations:** Department of Veterinary Medicine, Kangwon National University, Chuncheon-si 24341, Gangwon-do, Republic of Korea; freedoung@gmail.com (M.J.);

**Keywords:** trochlear block recession, medial patellar luxation, finite element analysis, fatigue analysis, trochlear groove depth, distal femoral fracture, mechanical testing

## Abstract

This study evaluated fracture risk associated with varying trochlear groove depth-to-patellar thickness (D/T) ratios in toy and small-breed dogs using finite element analysis and cadaveric mechanical testing. Finite element models derived from computed tomography data of a 4.5-kg toy poodle were adjusted to simulate D/T ratios ranging from 0.5 to 2.0 and analyzed for von Mises stress, principal strain, safety factor, and fatigue life under standing, trotting, and jumping conditions. Increasing D/T ratios led to progressive rises in stress and compressive strain, with the safety factor falling below 1.0 during jumping at ratios of 1.0 or higher. Fatigue life declined sharply beyond a ratio of 1.25 and reached zero-cycle failure at 2.0. Complementary mechanical testing of six distal femurs (6.42–8.7 kg), surgically modified to D/T ratios of 0.5, 1.0, or 1.5, revealed fracture patterns consistent with finite element predictions. These findings suggest that excessive trochlear deepening may compromise femoral integrity and elevate fracture risk, particularly under cyclic loading. Maintaining a D/T ratio between 0.75 and 1.0 may provide an optimal balance between effective patellar tracking and mechanical safety.

## Simple Summary

Trochlear block recession is a common surgical procedure used to treat Patellar Luxation in small dogs. However, if the groove in the distal femur is made too deep during surgery, it may weaken the distal femur and increase the risk of distal femoral fracture. This study aimed to evaluate how different groove depths affect the mechanical strength of the distal femur and its durability. Using computer-based modeling and laboratory testing of canine distal femurs, we found that deeper grooves led to higher stress within the distal femur and reduced its ability to withstand repeated loading, such as during activities like running or jumping. In particular, excessive deepening significantly increased the likelihood of structural damage and distal femoral fracture over time. Our results suggest that maintaining a moderate groove depth may help balance surgical effectiveness and preservation of the structural integrity of the distal femur. These findings provide practical guidance for veterinarians to reduce complications and improve surgical outcomes in small-breed dogs undergoing treatment for Patellar Luxation.

## 1. Introduction

Medial patellar luxation (MPL) is one of the most common developmental disorders causing hindlimb lameness in dogs [1,2]. It is particularly prevalent in toy and small breeds, whereas lateral luxation occurs more frequently in large breeds [3]. Numerous surgical techniques have been developed to correct MPL, with trochleoplasty being one of the most widely performed [4,5,6]. While clinical superiority over other forms of recession sulcoplasty has yet to be proven, trochlear block recession has been proposed, based on ex vivo studies, as advantageous for recessing a larger proportion of the trochlear surface and potentially improving resistance to patellar luxation [7,8]. In human medicine, trochleoplasty has been indicated for patellar instability to restore patellar tracking in line with the quadriceps mechanism and overall quadriceps alignment. However, direct extrapolation of these indications to canine patients remains limited [9].

Reported complications of trochleoplasty include recurrent luxation, displacement of the recessed block, fractures of the distal femur or trochlear ridge, and postoperative infections such as septic arthritis [10,11]. Excessive deepening of the trochlear groove has been linked to structural failure, including severe distal femoral fractures, whereas insufficient deepening may predispose to recurrence by failing to provide adequate patellar seating [10,11,12].

According to Slocum, a functional groove requires recessing approximately 50% of the patellar thickness [4,13]. More recent studies reported that the average trochlear groove depth in healthy adult dogs is about 46% of the patellar thickness [14]. Although these anatomical proportions have been established in normal dogs, quantitative evidence is still lacking to define the threshold at which surgically deepened grooves increase the risk of femoral fractures. Although distal femoral fracture following trochleoplasty has been reported infrequently in the veterinary literature, it is recognized as one of the major postoperative complications associated with excessive trochlear recession and technical error, emphasizing the importance of establishing biomechanically safe recession depths [10,11].

To address this gap, the present study quantitatively evaluates the fracture risk associated with surgically created groove depths using finite element analysis (FEA). Finite element models were developed to simulate stress distribution and fracture mechanics across varying groove depths. The computational findings were validated through mechanical testing of cadaveric femurs to determine whether the observed fracture locations and magnitudes corresponded with the predicted stress concentrations.

Finite element modeling has been validated for predicting bone stiffness and yield loads in three-point bending of canine long bones [15]. In this study, computed tomography (CT) data from a toy dog were used to construct anatomically accurate femoral models. Previous CT-based studies have demonstrated that canine femoral morphology, including femoral anteversion and neck angle, can be reliably quantified using three-dimensional imaging, supporting the use of CT-derived subject-specific models in biomechanical analyses [16]. Artificial modifications at varying recession depths were applied to replicate postoperative geometry, after which material properties were assigned based on CT-derived calibration and previously published canine bone literature, as described in the Materials and Methods. Meshes were then generated, and the models were analyzed to evaluate stress pathways and potential failure modes, with additional fatigue analysis performed to assess the effects of repetitive loading on structural integrity.

## 2. Materials and Methods

### 2.1. Development of Trochlear Block Recession Models

**Figure 1:**
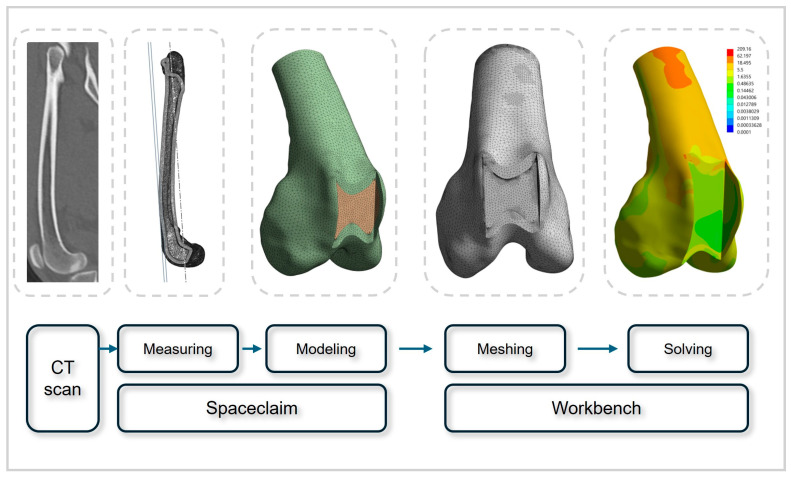
Workflow schematic illustrating the process leading to finite element modeling and analysis.

**Figure 2:**
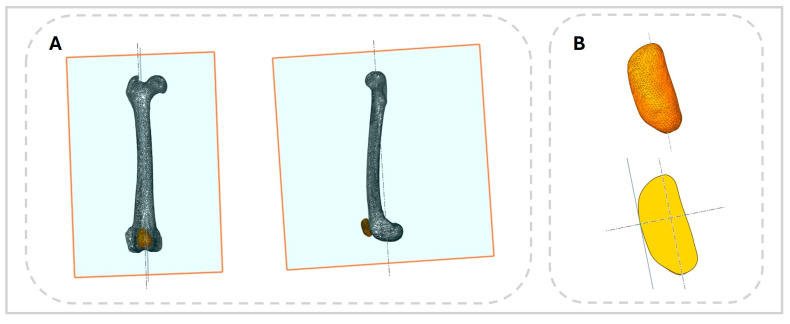
Anatomical measurements obtained with ANSYS SpaceClaim. (**A**) Sagittal and frontal planes parallel to the mechanical axis of the femur used for anatomical measurements. (**B**) Equivalent methodology applied to determine patellar length and thickness.

An MPL model was developed using CT data from a three-year-old toy poodle weighing 4.5 kg that had no orthopedic, neurological, or systemic disease. Imaging was performed with a 16-slice helical CT scanner (Alexion, Canon, Japan) at a slice thickness of 0.5 mm (Figure 1). CT data were imported into the 3D Slicer software (version 5.8.1, accessed on 5 April 2025, www.slicer.org), where the femur and patella were reconstructed into three-dimensional models and the surface mesh data were exported in STL format. These models were then imported into ANSYS SpaceClaim (version R2, Ansys Inc., Canonsburg, PA, USA), where anatomical measurements of the femur and patella were obtained and MPL models were created. To ensure consistency, sagittal and frontal planes were defined relative to the anatomical axis of the femur. Groove depth and femoral thickness were measured at the deepest point of the trochlear groove using these planes. For the patella, an axis was defined by connecting the cranial and caudal poles, and sagittal and frontal planes parallel to this axis were used to determine patellar thickness and length (Figure 2).

**Figure 3:**
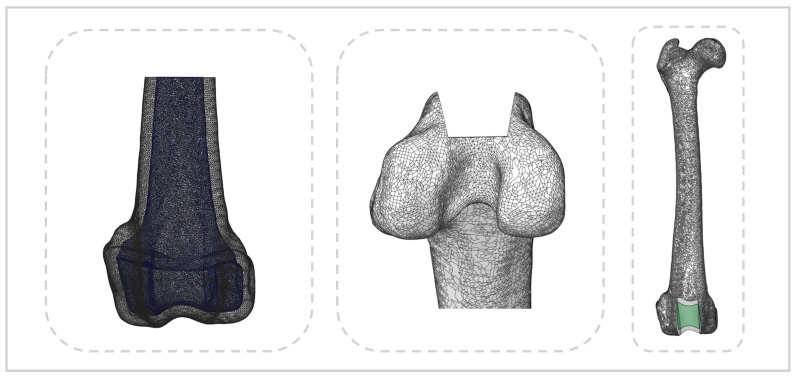
Medial patellar luxation model generated in ANSYS SpaceClaim. Cortical and trabecular bone of the femur are distinguished (left), and the block recession model is shown (middle and right).

Virtual trochlear recession models were constructed according to the surgical guidelines applied to cadaveric specimens. Seven models were generated with groove depths set at 0.5, 0.75, 1.0, 1.25, 1.5, 1.75, and 2.0 times the patellar thickness. In all models used for FEA, only the distal one-third of the femur was retained (Figure 3).

### 2.2. FEA

Meshing, material property assignment, and structural analysis were performed using the ANSYS Workbench software (version R2, Ansys Inc., Canonsburg, PA, USA). The material properties of the bone models are summarized in Table 1. Bone was assumed to behave as an isotropic, linearly elastic material [17,18]. Bone density (*ρ*) was calculated directly from CT-derived Hounsfield unit (HU) values using a previously validated conversion formula [15]. In contrast, Young’s modulus and Poisson’s ratio were assigned based on previously published finite element studies of the canine femur. The applied equation was:

*ρ* = 0.9839 + 0.00049332 × HU

**Figure 4:**
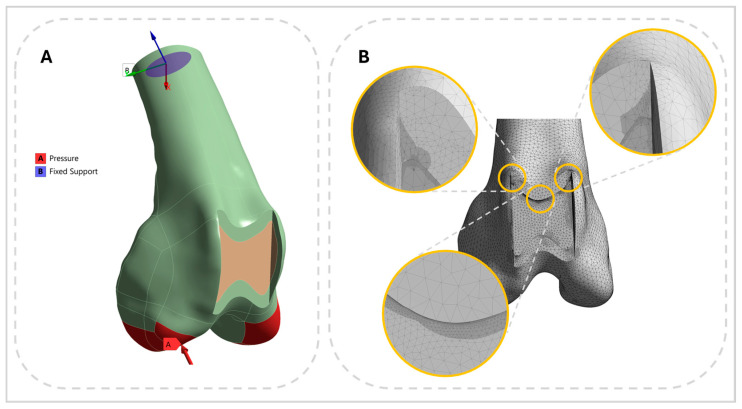
Pre-analysis configuration in ANSYS 480 Workbench. (**A**) The distal femur was fixed at the cut surface, and a vertical load was applied to the articular surface. (**B**) Mesh refinement applied to regions of high stress concentration or sensitivity to mesh quality.

The finite element mesh consisted of tetrahedral elements, with node counts ranging from 625,265 to 839,083 (median: 739,785). Regions subject to high stress or geometric complexity were refined locally to a mesh size of 0.025 mm (Figure 4), whereas other regions were meshed with element sizes between 0.4 and 0.6 mm. A convergence study was performed, and all elements maintained an aspect ratio below three. Boundary conditions were defined by fixing the proximal end of the femur model, while compressive loads were applied vertically to the distal articular surface of the condyles (Figure 4). Only axial compressive loading was considered, with magnitudes corresponding to the forces acting on a single hindlimb during different gait conditions in dogs: standing (18.5% of body weight), trotting (46%), and jumping (150% of body weight) loading conditions were applied. The jumping load was derived by converting peak vertical ground reaction forces reported during jump landing (expressed as N/kg body mass) to a percentage of body weight using a gravitational acceleration of 9.81 m/s^2^ [19,20,21].

### 2.3. Choice of the Failure Criterion

Fracture risk was assessed using a failure criterion derived from previously validated models [22]. Fracture potential at different groove depths was evaluated by calculating the risk for fracture (RF), defined as the ratio of the absolute value of the minimum principal strain to the ultimate compressive strain threshold (*ε_lim_*) of cortical bone:

$$RF=\left|\varepsilon_{min}\right|/\varepsilon_{lim}$$

Here, *|ε_min_|* represents the absolute value of the minimum principal strain. Because ultimate strain differs between tensile and compressive loading, the compressive strain limit was set to *ε_lim_* = 0.0104, consistent with previously reported experimental data for cortical bone [22]. An RF value greater than 1 was interpreted as indicating a potential fracture risk.

### 2.4. Fatigue Analysis

Fatigue analysis was conducted using ANSYS Workbench (Mechanical Module) to evaluate relative fatigue susceptibility of cortical bone under cyclic loading. The analysis followed a stress–life (S–N curve) approach. Representative alternating stress values were adopted to define the S–N relationship at the material level, rather than to justify the use of finite element modeling. Specifically, alternating stress levels of 83.39 MPa at 10^3^ cycles and 46.35 MPa at 10^6^ cycles were selected to represent low-cycle and high-cycle fatigue regimes, respectively. These values were used to define the upper and lower bounds of the fatigue life range and were interpolated on a log–log scale to construct the S–N curve employed in the present analysis [23,24].

The loading type was set to fully reversed cyclic loading. Mean stress correction was applied using the Goodman criterion, and von Mises equivalent stress was used as the stress component for fatigue evaluation. Fatigue life was defined as the number of cycles to failure at a given stress amplitude, damage as the accumulated fatigue damage fraction, and the safety factor as the ratio of allowable fatigue strength to the applied equivalent stress. Fatigue life, damage, and safety factor contours were calculated under steady-state loading conditions and post-processed to visualize fatigue-critical regions based on the number of cycles to failure.

### 2.5. Specimen Preparation

**Figure 5:**
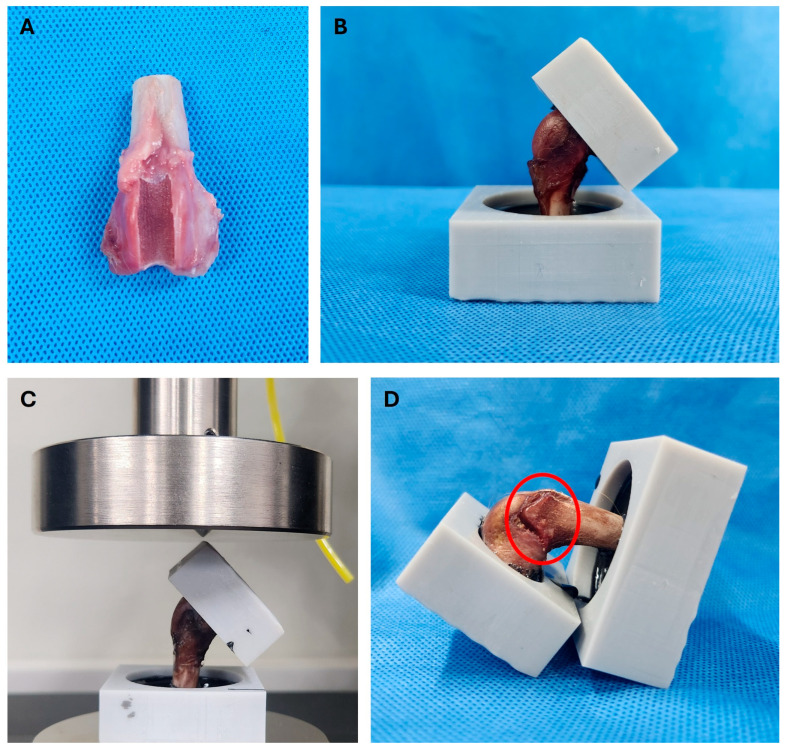
(**A**) Cadaveric femur specimen following trochlear block recession and osteotomy. (**B**) Fixation of the femoral shaft in a custom three-dimensionally printed mold using quick-cure resin. The femoral condyles and trochlear surface were not circumferentially embedded and remained free of lateral constraint to avoid buttressing effects. (**C**) Axial compression testing setup, in which a vertical compressive load was applied through a flat platen aligned with the global vertical axis. (**D**) Post-compression image showing the fracture site at the proximal trochlear region (red circle).

Femurs (right and left hindlimbs) were collected from three healthy mixed-breed dogs, each weighing less than 10 kg (median: 7.79 kg; range: 6.42–8.7 kg), that had been euthanized for reasons unrelated to this study. Although the exact ages of the cadaveric specimens were unavailable because they were obtained from institutional cadaver resources, all specimens were confirmed to be skeletally mature based on complete physeal closure. Cadavers were stored at −20 °C and thawed at room temperature for 24 h before dissection. Surgical correction of MPL was performed on each limb following a previously described protocol [12]. Briefly, a lateral parapatellar approach was used to incise the skin, fascia, and joint capsule, exposing the trochlear groove. Resection lines were marked immediately medial to the trochlear ridge, and abaxial cuts on both sides were angled approximately 10° axially toward the sagittal plane of the femur. The block length was designed to permit smooth patellar articulation within the joint. Recession depth varied by body weight category, with three groove depth conditions established: 0.5, 1.0, and 1.5 times the patellar thickness. Two femurs were prepared for each condition, yielding six specimens (Figure 5). After surgery, all surrounding soft tissues were removed, and only the distal one-third of each femur was retained. The femoral shafts were embedded in quick-cure resin (Smooth-Cast ONYX Fast; Smooth-On, Inc., Macungie, PA, USA) using custom three-dimensionally printed molds (Phrozen Mega 8 K, Phrozen, Hsinchu, Taiwan). These molds were designed to secure the femoral shaft and ensure that load was applied exclusively to the distal joint surface (Figure 5). To direct force specifically onto the articular surface, the upper boundary of the mold in contact with the joint was tapered to a sharp edge (Figure 5). The femoral shaft was rigidly embedded in resin for fixation, whereas the femoral condyles and trochlear surface were not circumferentially embedded and remained free of lateral constraint. The distal articular surface was oriented upward and placed in contact with a flat support surface of the mold, allowing controlled contact without providing a buttressing effect to the condyles. This resin-based fixation and alignment strategy was standardized across all specimens using identical custom molds and was conceptually consistent with previously reported cadaveric femoral mechanical testing setups employing non-102 coplanar distal fixation to ensure stable boundary conditions under axial compression [25]. Once cured, the specimens underwent mechanical testing. All procedures were approved by the Institutional Animal Care and Use Committee of Kangwon National University (Approval No. KW-250610-1). The femora used for ex vivo mechanical testing and those used for finite element model development were obtained from different animals.

### 2.6. Mechanical Testing

Each specimen was mounted on a universal material testing machine (AUTOGRAPH AGS-X; SHIMADZU, Kyoto, Japan), and axial compression testing was performed by applying a vertical compressive load through a flat platen aligned with the global vertical axis of the mold (Figure 5). The loading direction was determined to reproduce a standardized axial compression pathway through the distal femur while minimizing variability associated with specimen orientation. The femoral shaft was embedded in the custom mold with its longitudinal anatomical axis aligned to the global vertical axis, allowing the compressive load to be transmitted directly through the trochlear region and femoral condyles. Although physiological weight-bearing in vivo involves a combination of axial, shear, and rotational forces generated by joint motion and surrounding soft tissues, isolated axial compression was intentionally selected to provide a reproducible biomechanical model for comparing the relative effects of different trochlear groove depths under identical loading conditions. The loading rate was set at 5 mm/min. A preload of 3 N was first applied to establish consistent contact between the loading platen and the articular surface before displacement-controlled compression was initiated. This preload represented only approximately 4.8% of the standing load expected for the smallest specimen (6.42 kg; estimated single-limb standing load ≈ 11.6 N based on 18.5% body weight) and was not intended to simulate physiological weight-bearing. Compression was then continued until a total displacement of at least 5 mm was reached, and the maximum load recorded at the point of structural failure was defined as the load-to-failure.

## 3. Results

### 3.1. FEA and Fatigue Analysis

**Figure 6:**
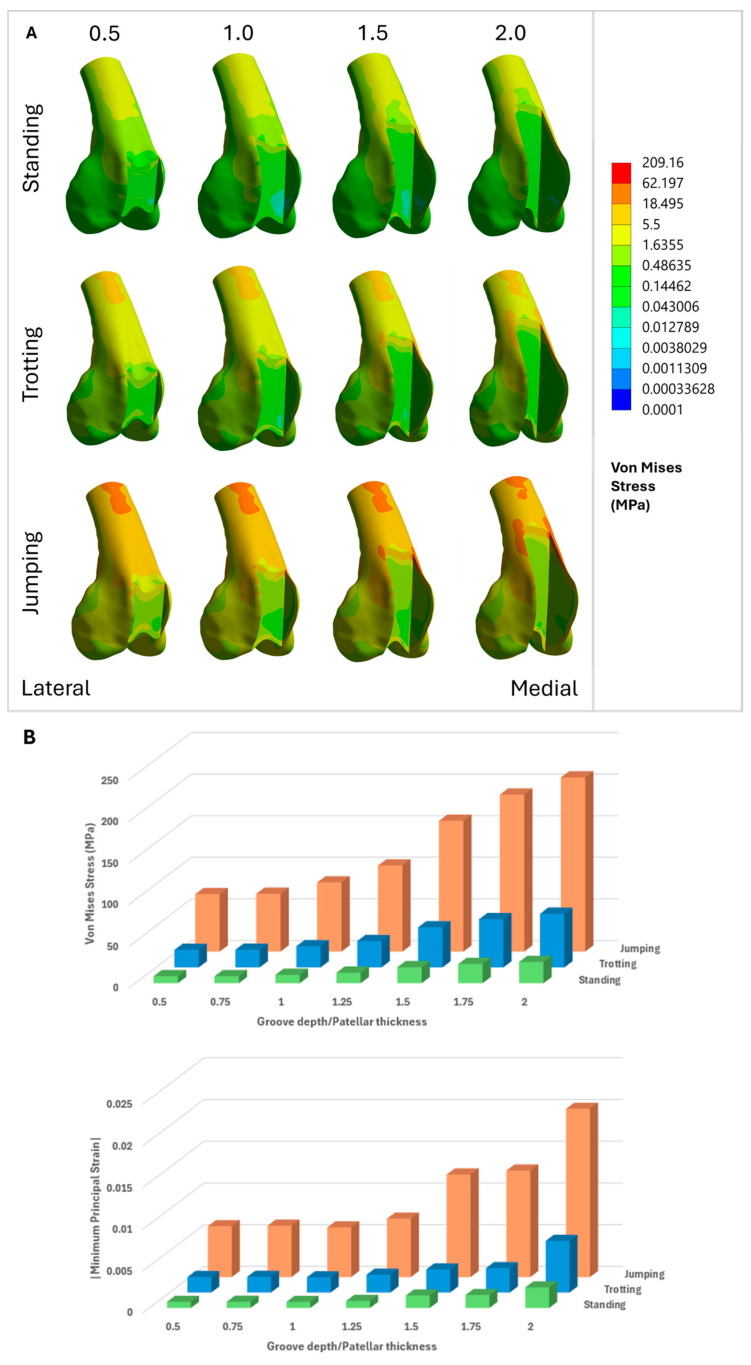
Visualization of finite element analysis (FEA) results. For all finite element models shown, the left side corresponds to the lateral aspect and the right side corresponds to the medial aspect of the distal femur. (**A**) Distribution of von Mises stress across varying D/T ratios for each gait pattern. (**B**) Graphs displaying the maximum von Mises stress (up) and the maximum absolute value of minimum principal strain (|Minimum Principal Strain|, down) according to D/T ratio for each gait pattern.

**Figure 7:**
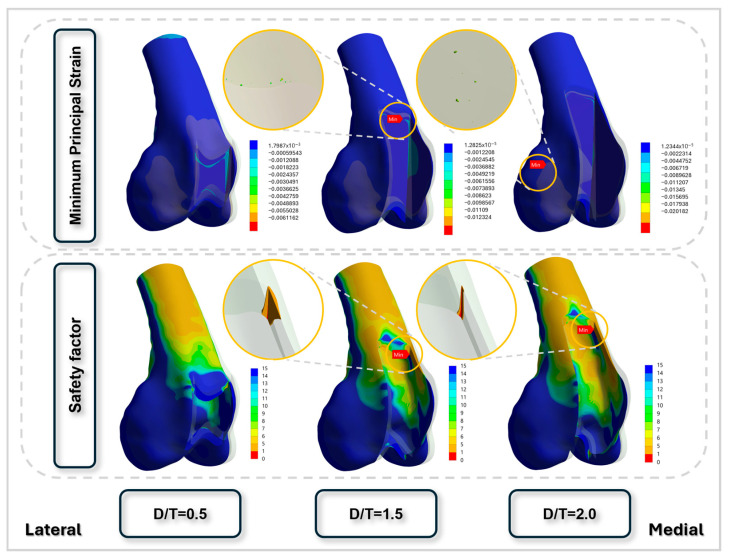
Visualization of peak strain distribution and regions most susceptible to fatigue fracture as the trochlear groove depth increases. For all finite element models shown, the left side corresponds to the lateral aspect and the right side corresponds to the medial aspect of the distal femur. At a D/T ratio of 1.5, the site of maximal strain coincides with the structurally weakest area. At a D/T ratio of 2.0, strain is predominantly concentrated in the mid-portion of the lateral condyle, while the zone of highest fatigue fracture susceptibility remains relatively constant.

FEA demonstrated distinct patterns of stress and strain distribution across the distal femur, which were strongly influenced by variations in the D/T ratio. Under all gait conditions (standing, trotting, and jumping), von Mises stress and minimum principal strain were primarily concentrated around the proximal medial ridge and its junction with the proximal trochlear groove, marking these areas as structurally vulnerable (Figure 6 and Figure 7).

As the D/T ratio increased, both peak stress and deformation rose markedly. At a ratio of 0.5, von Mises stress remained below 100 MPa under jumping conditions, whereas at a ratio of 2.0, stress peaked at approximately 209.16 MPa, representing more than a twofold increase. Under jumping conditions, localized peak von Mises stresses were also observed in the proximal diaphyseal region adjacent to the fixed boundary; however, these stress concentrations were consistently present across all D/T ratios and were attributed to boundary condition effects rather than changes in trochlear groove geometry. Minimum principal strain also intensified with higher ratios, indicating a greater likelihood of compressive failure in regions exposed to elevated mechanical demand (Figure 6).

RF, defined as the ratio of peak compressive strain to ultimate compressive strain (0.0104), increased sharply with both the D/T ratio and gait intensity. In this context, the safety factor corresponds to the inverse of the risk factor, whereas fatigue life denotes the estimated number of loading cycles until failure under the specified gait condition. Values greater than 1 indicated a substantial risk of fracture, particularly under high-impact loading such as jumping. The corresponding safety factor declined from 8.4 at a D/T ratio of 0.5 during standing to 0.358 at a ratio of 2.0 during jumping, highlighting the loss of structural integrity with excessive groove recession (Table 2).

Fatigue analysis revealed a clear inverse relationship between the D/T ratio and the fatigue life of the distal femur. Under cyclic loading conditions simulating gait-related joint forces, femurs with lower ratios demonstrated substantially longer fatigue lives and a reduced risk of cumulative damage. Specimens with ratios up to 1.25 generally exhibited fatigue lives exceeding 10^5^ to 10^6^ cycles, remaining below the strain-based compressive failure threshold applied in this study (Table 2). These configurations maintained low damage ratios and stable safety factors across all gait scenarios, indicating sufficient resistance to microstructural degradation under repeated loading.

In contrast, specimens with D/T ratios of 1.5 or greater showed a pronounced reduction in fatigue performance, particularly under simulated jumping conditions. At a ratio of 1.5, fatigue life dropped to near-immediate failure, whereas at 2.0 it declined to zero cycles (Table 2). Safety factors in these models fell below 1.0, and damage maps revealed highly localized stress and strain near the medial proximal trochlear ridge and lateral condylar regions (Figure 7). These areas corresponded to the initial fracture sites observed in the mechanical load-to-failure tests, reinforcing the predictive validity of the simulations.

### 3.2. Mechanical Testing

Following mechanical testing, the load-to-failure values were summarized according to the ratio of trochlear groove depth to patellar thickness (D/T) for each body weight group (Table 3). Fracture lines consistently originated at the junction of the proximal medial ridge and the proximal aspect of the trochlear groove, then propagated transversely across the femoral surface. The load-to-failure values represent the structural failure threshold of a single femur under isolated axial compression and are not intended to directly reflect steady-state in vivo weight-bearing conditions.

## 4. Discussion

This study examined the mechanical consequences of varying D/T ratios on the distal femur in toy and small-breed dogs by integrating cadaveric mechanical testing with computational FEA, including fatigue assessment. The results generally showed that higher ratios markedly compromised structural stability and fatigue resistance, particularly under simulated jumping loads. These findings suggest that deeper trochlear recession may increase fracture susceptibility under physiological loading conditions. Although postoperative activity restriction and cage confinement are routinely recommended following trochleoplasty, transient high-magnitude loading may still occur during unexpected movements such as jumping, slipping, or rapid deceleration, particularly in noncompliant patients, and may contribute to overload of a structurally weakened distal femur.

FEA demonstrated that von Mises stress, minimum principal strain, and localized deformation increased progressively with higher D/T ratios (Figure 6). Notably, fatigue life dropped to less than 10^5^ cycles at a D/T ratio of 1.0 under jump loading and reached zero cycles at 1.5 (Table 2). The fatigue life values presented in Table 2 represent the predicted number of loading cycles to structural failure under the simulated loading conditions and should be interpreted as a relative biomechanical index rather than an estimate of actual gait cycles or clinical lifespan. Accordingly, the fatigue life of 84,654 cycles observed at a D/T ratio of 1.0 under jumping conditions indicates substantially greater structural durability than deeper recession models but does not imply that fracture would occur after exactly 84,654 jumps in vivo. This sharp decline indicates markedly reduced structural durability under repetitive loading rather than predicting failure after a specific number of activities [26,27]. Fatigue damage maps revealed pronounced stress concentrations at the medial trochlear ridge and proximal trochlear groove (Figure 7), which corresponded to the fracture sites observed in mechanical testing, thereby reinforcing the predictive accuracy of FEA models. Furthermore, the safety factor fell below 1.0 at D/T ratios of 1.5 and 2.0 (Table 2), indicating that the applied stress exceeded the yield capacity of the bone and rendering fatigue failure inevitable [27,28].

These findings align with prior experimental and imaging-based reports suggesting that deep trochlear recession may introduce unintended cortical stress concentrations or produce non-physiological geometries [5,7,12], although few of these studies quantitatively evaluated fatigue life or structural failure. The present study is among the first to systematically integrate cadaveric data with FEA across different D/T ratios, demonstrating that fatigue failure may occur even in the absence of acute overload [19,29]. This insight is particularly important because fatigue-related failure may not be radiographically detectable until late in the degenerative process [23].

The clinical implication of these findings is that conservative groove recession, particularly maintaining D/T ratios between 0.75 and 1.0, may provide an optimal balance between restoring patellar tracking and preserving bone integrity. Ratios exceeding 1.25 were consistently associated with marked declines in fatigue performance and structural reliability. Although deeper grooves may seem to enhance patellar seating in the short term, they may paradoxically increase the risk of long-term failure by weakening subchondral support [8,30]. Therefore, preoperative planning should account not only for joint alignment but also for the mechanical consequences of D/T variation.

The cadaveric mechanical testing supported the computational predictions, showing that femurs with shallow grooves (D/T = 0.5) tolerated loads exceeding 2000 N, whereas those with deeper recessions (D/T ≥ 1.5) failed at loads below 200 N (Table 3). These load-to-failure values should not be interpreted as physiological standing loads but rather as the structural failure threshold of the surgically modified femur under isolated axial compression. This sharp reduction in mechanical strength indicates that excessive deepening of the trochlear groove can considerably weaken the bone, potentially increasing susceptibility to elevated mechanical loading under high-load conditions. In small-breed dogs, transient high-magnitude loads may occur during activities such as jumping or rapid deceleration, during which a substantial proportion of body weight can be concentrated on a single limb. Under such conditions, the reduced load-to-failure observed with increasing D/T ratios suggests increased susceptibility to single-event overload failure rather than normal steady-state weight bearing. Because small-breed dogs frequently require surgical management for medial patellar luxation [2,11], these biomechanical vulnerabilities should be carefully weighed when selecting surgical techniques. The proposed D/T ratio thresholds should be interpreted as relative biomechanical safety guidance rather than absolute fracture limits. The actual postoperative risk of distal femoral fracture is likely influenced by patient compliance, body weight, and postoperative activity level, with overweight dogs or those experiencing unexpected high-impact loading potentially being at greater risk despite routine exercise restriction. In vivo loading conditions include additional shear and torsional components that were not incorporated in the present model, and therefore clinical decision-making should consider these factors.

This study has several limitations that should be acknowledged. First, ex vivo mechanical testing and finite element analysis were performed on femora obtained from different animals. As such, the finite element analysis was not intended for direct one-to-one validation against experimental results, but rather to investigate relative trends in stress distribution and mechanical failure risk across varying D/T ratios. Second, bone material behavior was simplified using isotropic material properties derived from CT-based calibration and literature-based assumptions, which do not fully capture the intrinsic anisotropy and biological heterogeneity of bone tissue. Third, loading conditions in both the cadaveric mechanical testing and finite element analysis were simplified to axial vertical compression. In the mechanical test, the distal one-third of the femur was aligned with the global vertical axis to provide standardized and reproducible loading across specimens, whereas the finite element analysis applied vertical forces representing peak ground reaction loads during standing, trotting, and jumping. Although these simplified loading conditions facilitate direct comparison among different D/T ratios, they do not fully reproduce the complex multidirectional loading environment of the canine stifle during in vivo weight-bearing, which includes additional shear forces, torsional moments, muscle contraction, and ligamentous restraint. Finally, finite element modeling was based on a single CT-derived femoral geometry; inclusion of a larger, population-based dataset would improve the generalizability of the findings. Nonetheless, the convergence of cadaveric and computational findings reinforces the conclusion that higher D/T ratios present significant biomechanical risks. With the growing use of MPL corrective surgery in small-breed dogs, this study provides evidence-based guidance to help avoid overcorrection and subsequent structural compromise. Future research should incorporate patient-specific musculoskeletal simulations and nonlinear fatigue modeling to further refine risk prediction. In addition, the present cadaveric mechanical testing and finite element models did not incorporate periarticular soft tissues, including the joint capsule, ligaments, tendons, and surrounding musculature. These structures contribute to load sharing and joint stabilization in vivo and may reduce local stress concentrations around the distal femur. Therefore, the fracture risk predicted in this study may represent a conservative biomechanical scenario, and the actual clinical risk of distal femoral fracture may be lower than predicted because of the stabilizing effects of surrounding soft tissues. In addition, because an intact femoral control group without trochlear block recession was not included, the biomechanical effects attributable specifically to the surgical procedure could not be directly distinguished from the intrinsic mechanical behavior of the normal distal femur. Furthermore, because all cadaveric specimens were skeletally mature, the present findings cannot be directly extrapolated to immature dogs with open physes, in which fracture susceptibility may differ because of growth plate weakness.

## 5. Conclusions

This study evaluated the biomechanical effects of varying trochlear groove depth on femoral integrity in toy and small-breed dogs using finite element and fatigue analyses supported by cadaveric mechanical testing. The results demonstrated that increasing groove depth was associated with elevated stress concentrations, reduced fatigue life, and decreased safety margins, particularly under higher loading conditions. Notably, excessive trochlear deepening markedly increased the potential risk of structural failure over repetitive loading cycles. These findings suggest that moderate groove depths, approximately 0.75–1.0 times the patellar thickness, may provide a favorable balance between joint stability and preservation of the structural integrity of the distal femur. While the findings should be interpreted as relative biomechanical trends rather than absolute clinical thresholds, they offer practical guidance for surgical decision-making. Further studies incorporating more comprehensive loading conditions and subject-specific data are warranted to refine these recommendations.

## Figures and Tables

**Table 1:** Material properties assigned to cortical and cancellous bone in the finite element models. Bone density was calculated from CT-derived Hounsfield unit values, whereas Young’s modulus and Poisson’s ratio were assigned based on previously published canine femur finite element studies.

Component	Material Property Young’s Modulus (E) (MPa)	Poisson Ratio (ν)	Density (g/cm^3^)
Cortical bone	13,150	0.3	2.054
Cancellous bone	750	0.3	0.804

**Table 2:** Risk factor, minimum safety factor, and minimum fatigue life at each groove depth-to-patellar thickness ratio across different gait conditions. The safety factor represents the ratio of the allowable compressive strain threshold to the applied minimum principal strain, and fatigue life represents the predicted number of cycles to failure under cyclic loading. Fatigue life represents the predicted number of cycles to structural failure under the simulated loading conditions and should be interpreted as a relative biomechanical index rather than an estimate of clinical lifespan or gait cycles.

**Groove Depth/Patellar Thickness = 0.5**			
**Gait type**	**Risk factor**	**Safety factor (minimum)**	**Life (minimum)**
Standing	0.071	8.416	10^6^
Trotting	0.179	3.354	10^6^
Jumping	0.587	1.030	10^6^
**Groove Depth/Patellar Thickness = 0.75**			
**Gait type**	**Risk factor**	**Safety factor (minimum)**	**Life (minimum)**
Standing	0.072	8.359	10^6^
Trotting	0.181	3.331	10^6^
Jumping	0.592	1.023	10^6^
**Groove Depth/Patellar Thickness = 1**			
**Gait type**	**Risk factor**	**Safety factor (minimum)**	**Life (minimum)**
Standing	0.070	6.987	10^6^
Trotting	0.175	2.784	10^6^
Jumping	0.573	0.855	84,654
**Groove Depth/Patellar Thickness = 1.25**			
**Gait type**	**Risk factor**	**Safety factor (minimum)**	**Life (minimum)**
Standing	0.081	5.605	10^6^
Trotting	0.206	2.233	10^6^
Jumping	0.674	0.686	1982.1
**Groove Depth/Patellar Thickness = 1.5**			
**Gait type**	**Risk factor**	**Safety factor (minimum)**	**Life (minimum)**
Standing	0.144	3.685	10^6^
Trotting	0.363	1.468	10^6^
Jumping	1.182	0.451	0
**Groove Depth/Patellar Thickness = 1.75**			
**Gait type**	**Risk factor**	**Safety factor (minimum)**	**Life (minimum)**
Standing	0.15	3.068	10^6^
Trotting	0.281	1.222	10^6^
Jumping	1.227	0.375	0
**Groove Depth/Patellar Thickness = 2**			
**Gait type**	**Risk factor**	**Safety factor (minimum)**	**Life (minimum)**
Standing	0.237	2.766	10^6^
Trotting	0.596	1.102	10^6^
Jumping	1.940	0.358	0

**Table 3:** Mechanical test results for load-to-failure according to body weight and groove depth-to-patellar thickness ratio.

Test Condition		Load to Failure (N)
Body Weight (kg)	Groove Depth/Patellar Thickness	
6.42	0.5	2366.047
	1.5	148.2227
8.25	1.0	358.7
	1.5	184.2924
8.7	0.5	3026.09
	1.0	378.26

## Data Availability

The datasets supporting the conclusions of this article are included within the article. Processed finite element analysis data are available, while raw imaging data are not publicly available due to institutional restrictions.

## Abbreviations

D/T	trochlear groove depth-to-patellar thickness ratios
FE	finite element
MPL	Medial patellar luxation
CT	computed tomography
HU	Hounsfield Unit
RF	Risk for Fracture
S-N	stress–life curve
